# Highly stable magic angle spinning spherical rotors

**DOI:** 10.5194/mr-1-97-2020

**Published:** 2020-06-18

**Authors:** Thomas M. Osborn Popp, Alexander Däpp, Chukun Gao, Pin-Hui Chen, Lauren E. Price, Nicholas H. Alaniva, Alexander B. Barnes

**Affiliations:** Laboratory for Physical Chemistry, ETH Zürich, Vladimir-Prelog-Weg 2, 8093 Zürich, Switzerland

## Abstract

The use of spherical rotors for magic angle spinning offers a number of advantages, including improved sample exchange, efficient microwave coupling for dynamic nuclear polarization nuclear magnetic resonance (NMR) experiments, and, most significantly, high frequency and stable spinning with minimal risk of rotor crash. Here we demonstrate the simple retrofitting of a commercial NMR probe with MAS spheres for solid-state NMR. We analyze a series of turbine groove geometries to investigate the importance of the rotor surface for spinning performance. Of note, rotors lacking any surface modification spin rapidly and stably even without feedback control. The high stability of a spherical rotor about the magic angle is shown to be dependent on its inertia tensor rather than the presence of turbine grooves.

## 1 Introduction

Magic angle spinning (MAS) nuclear magnetic resonance (NMR) is usually used for high-resolution analysis of the local chemical environments of nuclear spins within biomolecular and inorganic solids [Bibr bib1.bibx22]. The sample is spun rapidly about an axis inclined at the magic angle, which is 54.74 ${}^{\circ }$ with respect to the external magnetic field $B_{0}$ . This averages terms in the NMR Hamiltonian whose orientational dependence is described by the second-order Legendre polynomial $3{\mathrm{cos}}^{2}\theta -1$ [Bibr bib1.bibx2]. For spins- 1/2 , MAS can yield spectra with highly resolved isotropic chemical shifts.

MAS has traditionally been performed by spinning a cylindrical rotor within a stator installed at the magic angle, thereby requiring a gas bearing to stabilize the rotor and drive gas to apply torque [Bibr bib1.bibx1]. However, we recently showed that it is possible to spin samples via a different paradigm, namely using spherical rotors spun using a single gas stream for both the bearing and drive [Bibr bib1.bibx6]. This approach allows highly stable rotor spinning about a single axis inclined at the magic angle, with record rates as high as 4.6 kHz ( $N_{2}$ , 4.1 bar) and 10.6 kHz ( He , 11 bar) for 9.5 mm diameter rotors and 11.4 kHz ( $N_{2}$ , 3.1 bar) and 28 kHz ( He , 7.6 bar) for 4 mm diameter rotors. Decreasing the rotor diameter permits even higher spinning rates. Additional benefits of spherical rotors include easy sample exchange and improved microwave access for dynamic nuclear polarization (DNP)–NMR experiments, yielding improved microwave $B_{1}$ field strength and homogeneity compared with current methods [Bibr bib1.bibx7].

In order to make spherical rotors robust and accessible for magnetic resonance experiments and to design apparatuses capable of achieving very high MAS rates, we examined the spinning and stabilization mechanisms of these spherical rotors. Here we (i) demonstrate how a stator for spinning spheres can be easily integrated into a commercial NMR probehead and (ii) examine the spinning behavior of a series of spherical rotors with various turbine groove geometries. We show that the spinning performance of spherical rotors can be improved by using a turbine groove geometry similarly to the drive tips used in conventional cylindrical rotor MAS systems, which are themselves based on the Pelton impulse turbine [Bibr bib1.bibx27]. However, we also find across a wide array of turbine styles that spinning performance is remarkably indifferent to the surface design and that even a rotor without turbine grooves can achieve stable, on-axis spinning. We show that a spherical rotor attains its stability from its inherent shape and mass distribution (i.e., its inertia tensor) and that turbine grooves are not essential for stable spinning.

## 2 Experimental apparatus

The experimental apparatus is depicted in Fig. [Fig Ch1.F1]. The stator employed for spinning spherical rotors was designed to adapt into a double-resonance APEX-style Chemagnetics probe (built decades ago for spinning 7 mm diameter cylindrical rotors) and 3D printed in clear acrylonitrile–butadiene–styrene (ABS) using either a ProJet MJP2500 3D printer (3D Systems, Rock Hill, SC, US) or a Form3 3D printer (Formlabs, Somerville, MA, US). A double-saddle coil made from 1.5 mm silver-coated copper magnet wire was wound by hand using a mandrel and wrapped in Teflon tape for insulation with the leads soldered into place in the existing radio frequency (RF) circuit of the Chemagnetics probe. The two optical fibers of the tachometer system were introduced into holes at the bottom of the semi-transparent stator. The transceiver of the original tachometer system was replaced with a more sensitive circuit. The transmitter in this circuit was an SFH756 light-emitting diode fed with 42 mA of current. The detector comprised an SFH250 photodiode and a transimpedance amplifier with a 4.7 MΩ feedback resistor followed by a gain block providing a voltage gain of 42. The magic angle adjustment was achieved by coupling the stator to the existing angle adjust rod in the probe.

**Figure 1 Ch1.F1:**
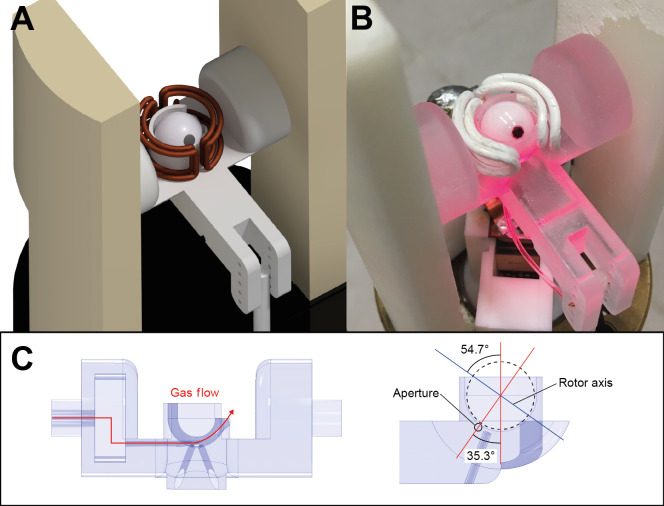
Experimental apparatus. **(a)** Probehead design schematic depicting the stator with a double-saddle coil, spherical rotor, and angle adjust arm. **(b)** Fully assembled probehead. **(c)** Cross-section views of the stator showing the drive gas inlet and angled through holes for the fiber optic tachometer. Drive gas is fed through the nozzle aperture placed at the complement of the magic angle (35.3 ${}^{\circ }$ ) into the stator cup.

NMR experiments were performed at 7.05 T using a Bruker Avance III spectrometer (Bruker Corp., Billerica, MA, US). ${}^{79}\mathrm{Br}$ spectra were taken at a transmitter frequency of 75.46 MHz and a MAS rate of 3.5 kHz. The implementation of a double-saddle coil within the probe enabled the application of a 35 kHz B ${}_{1}$ field to ${}^{79}\mathrm{Br}$ with 300 W incident RF power, a significant improvement over our previous implementation of 9.5 mm spherical rotors, which achieved a B ${}_{1}$ field of only 12.5 kHz using a split coil and 800 W incident RF power [Bibr bib1.bibx6].

The 9.5 mm spherical rotors were machined from yttria-stabilized zirconia (O'Keefe Ceramics, Woodland Park, CO, US). Seven new spherical rotor designs were introduced (Fig. [Fig Ch1.F2]), each with a 2.54 mm inner diameter cylindrical through hole: notched (rotors A, C), Pelton-style (rotors B, F), circular (rotors C, G), dimpled (rotor D), and with no flutes (rotor H). For the notched, circular, and Pelton-style rotors, two variations were machined differing by 0.5 mm in depth. For spin testing, each rotor was filled with a rigid 3D printed cylindrical ABS blank terminated with 4.75 mm spherical radius contoured ends. For the NMR experiment, the rotor was packed with 97.1 mg KBr powder and sealed at each end with 3D printed ABS plugs. The same stator design was used for all spin testing experiments.

**Figure 2 Ch1.F2:**
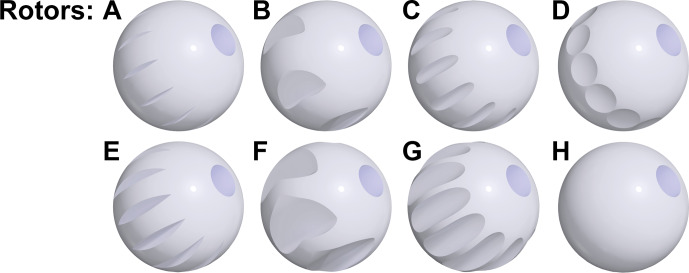
9.5 mm spherical rotor turbine groove designs. Each rotor has a 2.54 mm diameter through hole. A: 12 60 ${}^{\circ }$ notched grooves, 0.35 mm depth (as described previously by [Bibr bib1.bibx6]). B: 6 impeller-style grooves, 0.25 mm radius. C: 12 circular 0.25 mm radius grooves. D: 12 dimpled grooves, 4.75 mm radius dimples. E: 12 60 ${}^{\circ }$ notched grooves, 0.85 mm depth. F: 6 impeller-style grooves, 0.5 mm radius. G: 12 circular 0.5 mm radius grooves. H: smooth surface, no flutes machined.

## 3 Results and discussion

Spherical rotors spin within the hemispherical stator cup by the application of a gas stream along the rotor's equator from a converging nozzle tangent to the rotor surface [Bibr bib1.bibx6]. The nozzle aperture is placed at the complement of the magic angle (35.3 ${}^{\circ }$ ) in order to tilt the spinning axis of the rotor to a value near the magic angle (as shown in Fig. [Fig Ch1.F1]c). The rotor turbine grooves are intended to provide a means to efficiently couple the gas stream to the rotor surface, converting the kinetic energy of the fluid flow into rotational kinetic energy.

The designs depicted in Fig. [Fig Ch1.F2] were chosen to explore this concept. Intuition might suggest that deeper grooves allow for greater coupling to the gas stream due to the increased surface area perpendicular to the direction of fluid flow. However, the deep-grooved rotors E, F, and G could not spin stably under any of the conditions tested. It is possible that the large spaces created by the removal of material to make these deep grooves allow chaotic and turbulent flows to develop within the stator cup. The shallow-grooved rotors A, B, C, and D, as well as rotor H, achieved stable spinning. Figure [Fig Ch1.F3]a shows the spin test data in air at gauge pressures ranging from 0 to 4.3 bar for these five rotors. Across the five turbine geometries, the spinning rate increased non-linearly with respect to the applied pressure. The maximum spinning rates for the five rotors at the pressures tested ranged between 4 and 6 kHz in air, with Pelton-style rotor B having the highest maximum spinning rate of 5.7 kHz. Note that the maximum tested pressure was not limited by the rotors, as their spinning rates would be predicted to increase with increasing pressure, but rather the maximum pressure was limited to ∼4 bar due to safety concerns with regard to the connecting assemblies. Rotor B's maximum observed rate was a significant improvement over our previous maximum of 4.6 kHz for 9.5 mm rotors in air, which was achieved using rotor A at 4.1 bar [Bibr bib1.bibx6]. As rotor A also performed better in our current tests, with a maximum rate of 5.2 kHz at 4.1 bar, we attribute some of the performance gains to the higher-precision 3D printing of our latest stators. However, the further increase to 5.7 kHz with rotor B is likely to be due to the Pelton-style grooves more efficiently coupling to the fluid flow.

**Figure 3 Ch1.F3:**
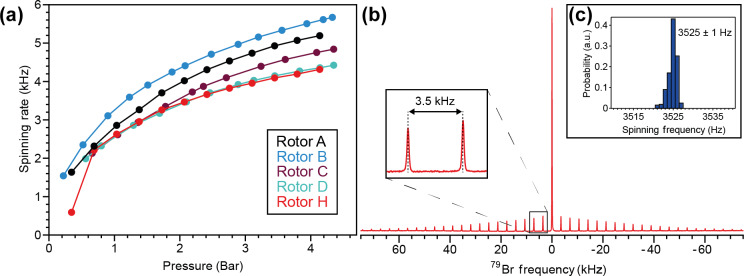
Spin test data. **(a)** Spinning rate as a function of applied air pressure for rotors A, B, C, D, and H. **(b)** ${}^{79}\mathrm{Br}$ spectrum of KBr packed into rotor H and spinning at 3.5 kHz at the magic angle; 512 scans. **(c)** Histogram of spinning frequencies without spinning regulation over the 10 min KBr data acquisition period.

A significant finding was that rotor H, with its smooth surface and lack of grooves, spun stably and on-axis. While not delivering the highest-frequency spinning of the tested rotors, its performance was comparable to many of the designs with machined grooves. Figure [Fig Ch1.F3]b shows the ${}^{79}\mathrm{Br}$ spectrum of KBr at 3.5 kHz MAS using rotor H. The stator's pitch angle was adjusted until the rotor's spinning axis was inclined to the magic angle, as observed by measuring the decay of rotor echoes out to 10 ms in the time domain data. The spectrum shows the spinning sideband manifold equally spaced by 3.5 kHz, corroborating the spinning rate observed by optical tachometry. The spinning was stable, with a standard deviation of ±1 Hz without the use of any spinning regulation mechanism (Fig. [Fig Ch1.F3]c). The ability of rotor H to spin stably at reasonable MAS rates implies that while turbine grooves can improve fluid flow and rotor coupling, a significant contribution to the overall spherical rotor spinning mechanism is simply from the torque created by drag induced by the driving gas stream moving across the rotor surface. Additionally, the fact that rotor H established a stable spinning axis about its own axis of symmetry shows that the grooves do not direct the rotor to spin about this axis, but rather that the geometry of the rotor itself must be responsible.

A spherical rotor with a cylindrical through hole is a solid known as a spherical ring. The moments of inertia for a spherical ring of constant density ρ , outer radius R , and inner radius r , where R≥r and z lies along the axis of symmetry, are given by

1Ixsr=Iysr=415πρR2-r23/24R2+r22,2Izsr=415πρR2-r23/22R2+3r2.

As most previous MAS experiments have been performed with cylindrical rotors, it is worth examining the inertia tensor of a cylindrical shell for comparison. For a cylindrical shell of constant density ρ , outer radius R , inner radius r , and length of 2kR , where k is the aspect ratio and R≥r , the moments of inertia are given by

3Ixcs=Iycs=πρkRR2-r2(2k+1)R2+r22,4Izcs=πρkRR2-r2R2+r2.

Figure [Fig Ch1.F4] shows the magnitudes of the moments of inertia for a spherical ring and a cylindrical shell ( k= 4) as a function of the inner radius given by Eqs. (1) and (2). For the spherical ring, $I_{z}$ is greater than or equal to the transverse moments $I_{x,\;y}$ for all values of r , while for the cylindrical shell, $I_{z}$ is less than or equal to the transverse moments $I_{x,\;y}$ for all values of r . When r=0 , the moments of inertia for the spherical ring and cylindrical shell are equivalent to those of a solid sphere and solid cylinder, respectively. Note that while Fig. [Fig Ch1.F4]b is representative of the high-aspect-ratio cylindrical rotors commonly used in MAS experiments, when k is low such that the geometry is disk-like, $I_{z}$ will be greater than $I_{x,\;y}$ for all values of r .

**Figure 4 Ch1.F4:**
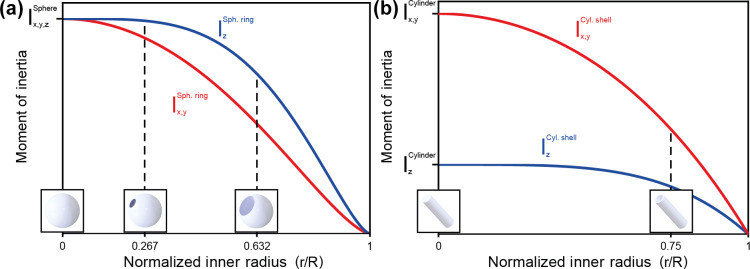
Moments of inertia for spherical and cylindrical rotors as a function of the normalized inner radius r/R . **(a)** $I_{z}$ (blue) and $I_{x,\;y}$ (red) for a spherical rotor as a function of the normalized inner radius. The rotors spun in this study correspond to r/R=0.267 . The difference between $I_{z}$ and $I_{x,\;y}$ is maximized at r/R=0.632 . **(b)** $I_{z}$ (blue) and $I_{x,\;y}$ (red) for a cylindrical rotor with aspect ratio k=4 as a function of the normalized inner radius.

Consider a rigid object with principal moments of inertia $I_{z}$ , $I_{y}$ , and $I_{x}$ , rotating about the axis coincident with $I_{z}$ with a constant angular velocity vector of $\left(\omega_{z},0,0\right)$ . If a small perturbation is applied, the angular velocity vector assumes the form $\left(\omega_{z},\lambda ,\mu \right)$ . Through substitution of this angular velocity vector into Euler's equations to yield a second-order linear differential equation with respect to λ [Bibr bib1.bibx24], we arrive at

5 $$\ddot{\lambda }+\frac{\left(I_{z}-I_{x})(I_{z}-I_{y}\right)}{I_{y}I_{x}}\omega_{z}^{2}\lambda =0.$$

Equation ([Disp-formula Ch1.E5]) can be used to arrive at the famous result of the intermediate axis theorem, which states that objects with $I_{z}>I_{y}>I_{x}$ will spin stably only about the first and third principal axes ( z and x ), while a rotation about the second principal axis ( y ) is unstable. However, for an axially symmetric object, where the axis of symmetry is aligned with the z axis and $I_{y}=I_{x}$ (such as a spherical ring or a cylindrical shell), Eq. (3) becomes

6 $$\ddot{\lambda }+\frac{(I_{z}-I_{x}{)}^{2}}{I_{x}^{2}}\omega_{z}^{2}\lambda =0.$$

Equation ([Disp-formula Ch1.E6]) implies that a rotation about the axis of symmetry ( z ) is always stable, as

7 $$\frac{(I_{z}-I_{x}{)}^{2}}{I_{x}^{2}}\omega_{z}^{2}>0.$$

If, however, the x axis is instead the axis of symmetry and $I_{z}=I_{y}$ , Eq. ([Disp-formula Ch1.E5]) reduces to $\ddot{\lambda }=0$ , whose solutions grow linearly in time. Thus, rotation about an axis perpendicular to the axis of symmetry is unstable. According to these results, we should expect that a rotation about the axis of symmetry ( z ) is stable and a rotation perpendicular to the axis of symmetry is unstable, regardless of whether $I_{z}$ is greater than or equal to $I_{x}$ .

From this result, we might expect that a spherical shell (axis of symmetry is the highest inertia axis) and a cylindrical shell (axis of symmetry is the lowest inertia axis) to both be continuously stable during a spin about their axis of symmetry. However, it has been observed experimentally that when avenues to dissipate energy are present, an axially symmetric object can dissipate energy to end up rotating about the axis that minimizes its rotational kinetic energy for a fixed angular momentum, which is the axis with the largest moment of inertia [Bibr bib1.bibx10]. This phenomenon has been observed in objects such as comets and asteroids as well as in early spacecraft such as Explorer 1 [Bibr bib1.bibx10]. Explorer 1 was a cylindrically symmetric satellite with a high aspect ratio that was meant to spin about its axis of symmetry (the lowest inertia axis) but ended up developing a precession as it transitioned into an end-over-end spin (the highest inertia axis) as a result of energy being dissipated into the structure.

Due to dissipative interactions with the surrounding fluid, a high-aspect-ratio cylindrical MAS rotor cannot spin stably and continuously about its axis of symmetry without active stabilization (i.e., bearing), as its symmetry axis is also its lowest inertia axis. However, in the case of a spherical MAS rotor, the rotor is placed in the stator without a specific orientation and initially may spin about an arbitrary axis. As the axis of symmetry for a spherical ring is also its greatest inertia axis, a spherical rotor ultimately ends up stably spinning about its axis of symmetry due to rotational energy being dissipated by interactions with the gas stream and surrounding atmosphere. For this reason, a spherical rotor shows very stable on-axis spinning and resilience to crashing, as the rotor will always return to a stable minimum about its axis of symmetry after a perturbation.

Equations (1) and (2) suggest that a spherical rotor should spin most stably about its axis of symmetry, z , when r/R=0.632 , where the difference between $I_{z}$ and $I_{x,\;y}$ is maximized (Fig. [Fig Ch1.F4]a). Critically, this means that rotors with a spherical ring geometry can be quite stable even with very large sample volumes. When considering a packed rotor, as long as the density of the caps and sample is lower than the density of the rotor material, $I_{z}$ will be greater than $I_{x,\;y}$ for all values of r/R between 0 and 1, and stable on-axis spinning will occur. A detailed investigation of the moments of inertia for a packed spherical rotor taking into consideration the caps, sample, and rotor, each with different densities, can be found as an interactive notebook in the Supplement.

## 4 Conclusions

While turbine grooves can help to increase MAS rates for spherical rotors, the inertia tensor is responsible for its spinning stability. As MAS rates continue to increase to values in excess of 100 kHz, the possibility of spinning instabilities leading to rotor crashes becomes a significant concern. Spherical rotors spinning at high rates will be able to self-correct to a stable state after a perturbation due to the highest moment of inertia axis being aligned with the axis of symmetry. To achieve high MAS rates with spherical rotors, new rotors with smaller outer diameters must be designed and fabricated. These rotors could use shallow, Pelton-style grooves to increase the maximum spinning rate by about 30 % compared to a rotor with no machined grooves, as observed here. However, since turbine grooves are not necessary to achieve stable spinning, these rotors could be fabricated without the need for complex micro-machining techniques to produce turbine grooves.

## Supplement

10.5194/mr-1-97-2020-supplement The supplement related to this article is available online at: https://doi.org/10.5194/mr-1-97-2020-supplement.

## Code availability

The Mathematica code used to produce the plots seen in Fig. 4 as well as an analysis of the inertia tensor as a function of normalized inner radius for a packed spherical rotor with unique densities for the rotor, sample, and caps may be found in the Supplement.
