# Supplementary material for: Highly stable magic angle spinning spherical rotors
Source: Magn Reson (Gott). 2020 Jun 18;1(1):97–103. doi: 10.5194/mr-1-97-2020 (PMC10500705; doi:10.5194/mr-1-97-2020)
Supplement: The supplement related to this article is available online at: https://doi.org/10.5194/mr-1-97-2020-supplement. [file mr-1-97-supplement.zip › mr-1-97-2020-supplement-title-page.pdf]

Supplement of Magn. Reson., 1, 97–103, 2020
https://doi.org/10.5194/mr-1-97-2020-supplement
© Author(s) 2020. This work is distributed under
the Creative Commons Attribution 4.0 License.

Open Access
MAGNETIC RESONANCE

Supplement of

Highly stable magic angle spinning spherical rotors

Thomas M. Osborn Popp et al.

Correspondence to: Alexander Barnes (alexander.barnes@phys.chem.ethz.ch)

- mr-1-97-2020-supplement-title-page.pdf
- supplementary_material_mathematica.nb
- supplementary_material_mathematica_pdfpreview.pdf

The copyright of individual parts of the supplement might differ from the CC BY 4.0 License.
