# Supplementary material for: Highly stable magic angle spinning spherical rotors
Source: Magn Reson (Gott). 2020 Jun 18;1(1):97–103. doi: 10.5194/mr-1-97-2020 (PMC10500705; doi:10.5194/mr-1-97-2020)
Supplement: The supplement related to this article is available online at: https://doi.org/10.5194/mr-1-97-2020-supplement. [file mr-1-97-supplement.zip › supplementary_material_mathematica_pdfpreview.pdf]

Understanding the effect of material densities on the moments of inertia for a packed spherical rotor

Supplementary interactive document to “Highly Stable Magic Angle Spinning Spherical Rotors Lacking Turbine Grooves”

Inertia tensor of a packed rotor

Model of an idealized “packed rotor”

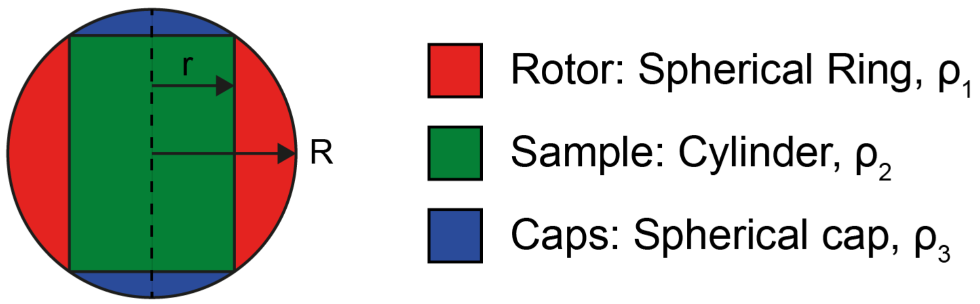

Since the inertia tensor of the spherical rotor is central to its spinning stability, it is worth examining the inertia tensor of a packed rotor containing a sample and caps with densities that differ from that of the rotor body. The figure above depicts a cross section of the model we will use, which contains three sections of differing density: the rotor (spherical ring), the sample (cylinder) and the caps (spherical caps). In reality, the caps would need to extend a little bit into the cylindrical sample space in order to stay in place, but with this idealized model one can clearly see the major effects of changing the density of the caps.

Generally speaking, the material used for these caps will likely be some sort of plastic (0.9-1.3 g/cm^3) or teflon (2.2 g/cm^3), which are significantly less dense than the most commonly used rotor material, zirconia (5.68 g/cm^3). Biological samples will have densities on the order of 1-2

g/cm^3, meaning that for most biological solid state NMR applications, the caps and sample will be of similar density and both will be less than the density of the rotor. Few NMR samples are much more dense than zirconia, but there are some materials like some heavy metal halide salts or ceramics which may be comparable or greater in density than zirconia. We could also potentially choose different rotor materials with lower density (nylon, alumina, silicon nitride), which while attractive to reduce the overall mass of the rotor, could also potentially be problematic for spinning stability. With our model, we can examine what will happen to the moments of inertia for the packed rotor in these cases.

Inertia tensor for a spherical ring

Inertia tensor for a cylindrical shell/ cylinder (r = 0)

Inertia tensor for the caps

Inertia tensor for a packed rotor, where the density of the rotor is ρ_1, density of the cylindrical sample space is ρ_2, and density of the caps is ρ_3:

Plot

Plot initialization

Plot with sliders

In[28]:=
```
Manipulate[
 Plot[{Iprx[1, r, ρ1, ρ2, ρ3], Iprz[1, r, ρ1, ρ2, ρ3]}, {r, 0, 1},
  PlotRange → {0, Isph[1, 1] * 1.05}, PlotStyle → {Red, Blue},
  Frame → True, FrameTicks → {{yticks1, False}, {True, False}},
  FrameLabel → {"Normalized inner radius, r/R", "Moment of inertia"}],
 {ρ1, 0, 1}, {ρ2, 0, 1}, {ρ3, 0, 1}]
```

Out[28]=

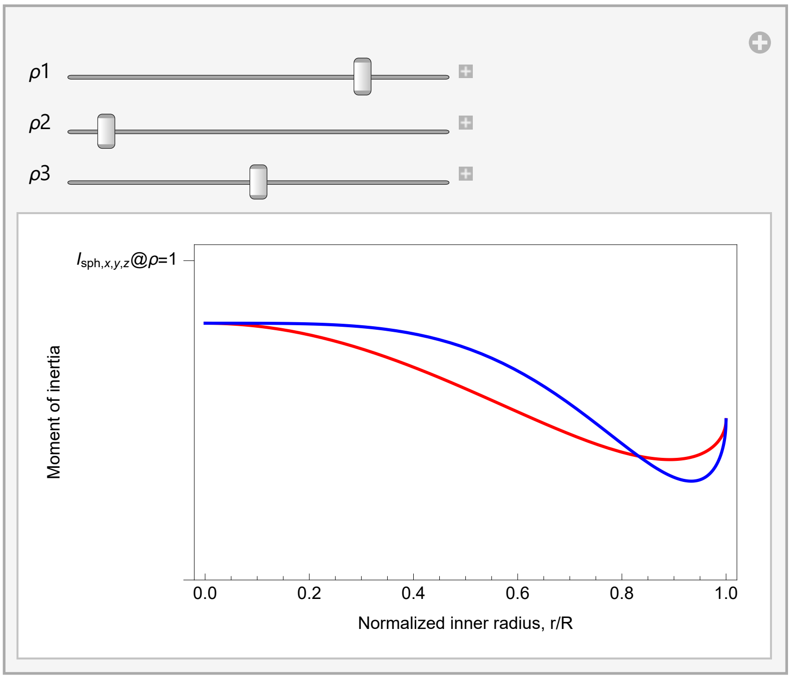

In cases where I_z (blue) > $I_{x,y}$ (red), we expect that the rotor should spin stably along its axis of symmetry. As you adjust the sliders, notice the wide range of density cases where this condition is satisfied. The most noticeable case where this condition is satisfied is when $\rho_1 > \rho_2 \geq \rho_3$. Even when $\rho_1 > \rho_2 < \rho_3$, only at large inner radius values do we see an inversion of the values of the moments. Generally, when ρ_1 is lower than ρ_2 or ρ_3, we see inversion over a wide range of inner radius values. However, interestingly, when $\rho_2 > \rho_1 \leq \rho_3$, the stable spinning condition is met at large inner radius values, suggesting that it is possible to spin high density samples as long as the inner diameter of the rotor is wide. This makes the mass distribution disk-like, and this makes sense that it would spin stably, as we are intuitively familiar with disks spinning stably about their “wheel axle” axis.
